# Vasorelaxant-Mediated Antihypertensive Effect of the Leaf Aqueous Extract from *Stephania abyssinica* (Dillon & A. Rich) Walp (Menispermaceae) in Rat

**DOI:** 10.1155/2021/4730341

**Published:** 2021-10-08

**Authors:** Chamberlin Fodem, Elvine Pami Nguelefack-Mbuyo, Magloire Kanyou Ndjenda II, Albert Kamanyi, Télesphore Benoit Nguelefack

**Affiliations:** Research Unit of Neuro-Inflammation and Cardiovascular Pharmacology, Faculty of Science, University of Dschang, P.O. Box 67 Dschang, Cameroon

## Abstract

*Stephania abyssinica* is a medicinal plant used in Cameroon alternative medicine to treat arterial hypertension (AHT). Previous *in vitro* studies demonstrated the endothelium nitric oxide-independent vasorelaxant property of the aqueous extract from *Stephania abyssinica* (AESA). But its effect on AHT is unknown. The present study was undertaken to explore other vasorelaxant mechanisms and to determine the antihypertensive effects of AESA in male Wistar rats. Phytochemical analysis of AESA was carried out using the liquid chromatography-mass spectrometry (LC-MS) method. The vasorelaxant effects of AESA (1-1000 *μ*g/mL) were studied on rat isolated thoracic aorta rings, in the absence or presence of indomethacin (10 *μ*M) or methylene blue (10 *μ*M). The inhibitory effect of AESA on phenylephrine (PE, 10 *μ*M) or KCl- (60 mM) induced contraction as well as the intracellular calcium release was also evaluated. The *in vivo* antihypertensive activity of AESA (43, 86, or 172 mg/kg/day) or captopril (20 mg/kg/day) administered orally was assessed in L-NAME- (40 mg/kg/day) treated rats. Blood pressure and heart rate (HR) were measured at the end of each week while serum or urinary nitric oxide (NO), creatinine, and glomerular filtration rate (GFR) were determined at the end of the 6 weeks of treatment, as well as histological analysis of the heart and the kidney. The LC-MS profiling of AESA identified 9 compounds including 7 alkaloids. AESA produced a concentration-dependent relaxation on contraction induced either by PE and KCl, which was significantly reduced in endothelium-denuded vessels, as well as in vessels pretreated with indomethacin and methylene blue. Moreover, AESA inhibited the intracellular Ca^2+^ release-induced contraction. In vivo, AESA reduced the AHT, heart rate (HR), and ventricular hypertrophy and increased serum NO, urine creatinine, and GFR. AESA also ameliorated heart and kidney lesions as compared to the L-NAME group. These findings supported the use of AESA as a potential antihypertensive drug.

## 1. Introduction

Cardiovascular diseases (CVDs) represent the first cause of death globally and account for approximately 18.6 million deaths each year making nearly 32% of all global deaths worldwide [1, 2]. Of these deaths, 10.8 million (19.2% of all deaths) are due to complications of arterial hypertension [3]. Arterial hypertension (AHT) affects 31.1% of adults (1.39 billion) worldwide [4]. It is the leading cause of CVDs and then responsible for 13% of premature deaths in developed and developing countries [5]. Recently, the definition of AHT has shifted from 140/90 mmHg to 130/80 mmHg for systolic/diastolic blood pressure, making almost half of the adult population hypertensive [6]. Although efforts have been made to reduce the burden of AHT, more and more people are diagnosed with AHT with a drastic increase in low- and middle-income countries including sub-Saharan African countries. AHT is, therefore, a real public health challenge. In sub-Saharan Africa, the prevalence of AHT has reached 25.4% and it is projected that by the year 2030, a 66% increase in AHT prevalence will be recorded if adequate measures are not taken [7]. AHT is particularly severe in African descent in whom a rapid onset, poor control, and early end-stage organ damage are observed [8, 9]. Poor AHT control leads to drastic outcomes including kidney failure, coronary heart disease, atherosclerosis, myocardial infarction, stroke, blindness and premature mortality, and disability [10, 11].

The main feature of essential hypertension which makes up to 90 to 95% of all AHT cases [12, 13] is increased vascular resistance due to imbalance between vasoconstricting and vasodilating substances produced by the endothelium known as endothelial dysfunction [14–16]. Thus, targeting endothelial dysfunction appeared as a good therapeutic option. Endothelial dysfunction can be induced experimentally by blocking the production of nitric oxide (NO), the main endothelial vasodilating factor using N*^ω^*-Nitro-L-Arginine Methyl Ester (L-NAME). This model is well accepted by the scientific community as it mimics hypertension in humans [17, 18]. Chronic administration of L-NAME has been associated with structural, functional, and biochemical alterations at the level of the heart, aorta, and kidney [18–20].

Although many antihypertensive drugs have been manufactured, the effective control of AHT is poorly achieved in patients from low- and middle-income countries due to several limitations such as resistance to therapy, inaccessibility, toxicity, high cost, and low compliance [21–23]. The search for new drugs, especially biological active compounds from natural sources, is of great interest for the development of low cost, more efficient, lesser side effect, and better-tolerated medication. Complementary and alternative therapies or pharmacological validation of ethnomedical medicine could greatly benefit patients in poor economic situations [24, 25].

Natural substances with vasorelaxant activities have been the focus of studies in the last decades and are recognized for their efficacy in preventing and treating hypertension [24, 26]. *Stephania abyssinica* (Dillon & A. Rich) Walp (Menispermaceae) is a twining liana rich in bioactive alkaloids, flavonoids, lignans, steroids, terpenoids, and coumarins [27–29].

The plant is widely used in African folk medicine for the treatment of various ailments including asthma, hyperglycemia, sleep disturbances, inflammation, men impotence, miscarriage, and liver diseases [28, 30–34]. *S. abyssinica* is also used to treat heart complaints [35], and in the West region of Cameroon, the aqueous extract from the fresh leaves of *S. abyssinica* is administered orally for the management of cardiovascular disorders including arterial hypertension. Previous *in vitro* studies demonstrated that the aqueous extract from *S. abyssinica* possesses endothelium nitric oxide-independent vasorelaxant effects [14]. However, other vasorelaxant mechanisms were still to be determined as well as the effect of *S. abyssinica* extract on the cardiovascular system. Therefore, the purpose of this study was to explore the antihypertensive properties of the aqueous extract from *S. abyssinica* in the L-NAME-induced hypertensive rat model and determine other vasorelaxant mechanisms and the protecting effect of the extract on selected target organs, assuming that this might contribute to the antihypertensive effect of the plant extract.

## 2. Materials and Methods

### 2.1. Drugs and Chemicals

Calcium chloride (CaCl_2_), N-(1-naphthyl)-ethylenediamine dihydrochloride (NED), magnesium sulfate (MgSO_4_), potassium chloride (KCl), sodium hydrogenocarbonate (NaHCO_3_), magnesium chloride (MgCl_2_), and methylene blue were purchased from MERCK (Germany). Sodium chloride (NaCl), dihydrogen phosphate (H_2_PO_4_), and D(+)-glucose were purchased from ROTH (Germany). Ethylenediaminetetraacetic acid (EDTA) was purchased from Fluka Chemika (Switzerland). Disodium hydrogen phosphate (Na_2_HPO_4_) was provided by Riedel de Haën AG. L-NAME, indomethacin, carbachol, creatinine, captopril, and phenylephrine were purchased from Sigma-Aldrich (Taufkirchen, Germany).

### 2.2. Plant Material and Extraction

The plant material collected in the West of Cameroon (Foréké-Dschang) in April 2018 was identified in Cameroon National Herbarium under the voucher specimen number 542/HNC. The aqueous extract was prepared using the protocol previously described by [14]. Fresh leaves of *S. abyssinica* (1 kg) were crushed twice in 2.5 L distilled water giving 5 L of solution. The solution obtained was filtered with Whatman No. 3 filter paper and lyophilized. This process yielded 47.4 g (4.74%) of dry powder which was stored at 4°C until use. For the in vitro experimentations, the powder (700 mg) was dissolved in 10 mL of distilled water to give a stock solution (70 mg/mL). For the *in vivo* experimentations, the powder (0.172 g) was dissolved in 10 mL distilled water to give a stock solution (172 mg/kg pc). Further dilution was made from stock solutions as needed.

### 2.3. Liquid Chromatography-Mass Spectrometry Profiling of the *Stephania abyssinica* Leaf Aqueous Extract

The phytochemical profiling of the aqueous extract from the leaves of *Stephania abyssinica* was carried out using liquid chromatography-mass spectrometry (LC-MS) technique [36]. The high-resolution mass spectrum was obtained using a QTOF spectrometer (Bruker, Germany) equipped with a hot electrospray ionization source. It was set in positive mode (mass range: 100-1500, with a scan speed of 1.00 Hz) with automatic gain control to provide high-accuracy mass measurements with a deviation of 0.40 ppm using sodium formate as a calibrant. A spray voltage of 4.5 kV and a capillary temperature of 200°C were used, with nitrogen as a gas sheath (10 L/min).

The spectrometer was connected to a UHPLC Ultimate 3000 system (Thermo Fisher, USA) consisting of an LC pump, an iodine detector array (DAD) (*λ*: 190-600 nm), an automatic injector sample (10 *μ*L), and a heating column (40°C). Separations were performed using Synergi MAX-RP 100A (50 × 2 mm; particle size 2.5 *μ*m) with a gradient of H_2_O (+0.1% HCOOH) (A)/acetonitrile (+0.1% HCOOH) (B) (circulation speed 500 *μ*L/min, injection volume 20 *μ*L).

The sample was analyzed using a gradient programmed as follows: 95% A isocratic (1.5 minutes) and linear gradient up to 100% B (6 minutes). After 2 minutes of 100% B isocratic, the system was returned to its initial condition (90% A) (1 minute) and was equilibrated for 1 minute.

The raw formulas, obtained from LC-MS, were first introduced into the PubMed, Google Scholar, and Scopus search engines to seek publications on the phytochemistry of *Stephania abyssinica* and to a certain extent on Menispermaceae. Secondly, the molecular formulas were introduced into the PubChem and ChemSpider search engines to obtain different nomenclature corresponding to each of the formulas. The names obtained were entered once again in the PubMed, Google Scholar, and Scopus engines in combination with the name of the plant or the family. Compounds that had a correspondence in these publications were identified as such.

### 2.4. Animal Housing

Wistar rats of both sexes aged 8-10 weeks and weighing 150 to 200 g were randomly selected from our colony. They were raised in the animal house of the Faculty of Sciences, University of Dschang, Cameroon, in plastic cages. Rats were housed 4 per cage and exposed to a natural light-dark cycle (~12 h/12 h). They were maintained at a room temperature of 22 ± 3°C with free access to food and water *ad libitum*. Experimental protocols used in this study were approved by the Laboratory Committee (Laboratory of Animal Physiology and Phytopharmacology, Department of Animal Biology, University of Dschang, Cameroon) according to the standard ethical guidelines for laboratory animal use and care as described by the law 2010/63/EU of the European Parliament and of the Council of 22 September 2010 on the protection of animals used for scientific purposes.

### 2.5. Screening of the Vasorelaxant Mechanism of the Aqueous Extract from *S. abyssinica*

#### 2.5.1. Aorta Isolation and Mounting

The aorta rings were prepared as previously described by Nguelefack et al. [14] and suspended at a resting force of 2 g in an oxygenated tissue bath (95% O_2_, 5% CO_2_) containing 10 mL Krebs solution, maintained at 37°C and pH 7.4. Changes in force were recorded isometrically using a force transducer connected to a kymograph with a data acquisition software (SPELL Advanced Kymograph Data Acquisition software, MDE Heidelberg). Aortic rings were equilibrated for 60 min during which the solution was renewed every 15 min.

#### 2.5.2. Elucidation of the Vasorelaxant Mechanisms of the Aqueous Extract from *S. abyssinica*

After the equilibration period, the functional integrity of the endothelium was assessed as follows: the aortic rings were precontracted with 10 *μ*M phenylephrine (PE), and when the contraction reached a plateau, carbachol (10^−5^ M) was added. The presence of endothelium was evidenced by a relaxation of at least 60% and considered destroyed when carbachol induced less than 10% relaxation. After this, the effect of cumulative concentrations of the aqueous extract of *S. abyssinica* (10-1000 *μ*g/mL) was tested on endothelium-intact aortic rings precontracted with PE or KCl (60 mM) in the presence or absence of indomethacin (10 *μ*M), a cyclooxygenase inhibitor, and methylene blue (10 *μ*M), a guanylate cyclase inhibitor. Rings were preincubated with each inhibitor for 20 minutes prior to precontraction with PE [14]. To evaluate the nontoxic effect of AESA, aortic rings were subjected to KCl or PE contraction after being exposed to the plant extract.

The effect of AESA on intracellular calcium was investigated as described by Chen et al. [24] with slight modifications. Briefly, after 1-hour stabilization period in normal Krebs solution, endothelium-denuded aortic rings were contracted with KCl. When the contraction reaches a plateau, the rings were washed in normal Krebs for 15 minutes and then transferred into Ca^2+^-free Krebs (+1 mM EDTA) for another 15 minutes. PE (10 *μ*M) was then added to induce the first transient contraction (T1). Following another washing in normal Krebs for 15 minutes and into Ca^2+^-free Krebs for another 15 minutes, rings were then incubated in the absence or presence of AESA (100 and 300 *μ*g/mL) for 20 minutes before the second transient contraction (T2) induced by PE (10 *μ*M). T1 and T2 were expressed as a percentage of the contraction induced by 60 mM KCl in normal Krebs, and the ratio of the second transient contraction to the first (T2/T1) was calculated.

### 2.6. Evaluation of the Antihypertensive Effect of the Aqueous Extract of *S. abyssinica*

#### 2.6.1. Animal Grouping and Dosing

Before the beginning of the experiment, male rats were acclimatized to the indirect blood pressure and heart rate recording using the tail-cuff plethysmography method (IITC Life Science, Woodland Hills, CA, USA). After measuring the baseline value for blood pressure and heart rate, rats were randomly assigned to two lots: lot A (normal control) (*n* = 8) and lot B (L-NAME hypertensive rats). Lot A received distilled water (10 mL/kg) while lot B was chronically administered with L-NAME (40 mg/kg pc/day) once daily for 3 consecutive weeks. At the end of the third week, rats of lot A became the normal control (group 1) and continue receiving distilled water; animals of lot B were distributed into 5 groups (groups 2 to 6) and treated for 3 other consecutive weeks as follows:

- Group 1: normal control rats administered only DW
- Group 2: L-NAME (40 mg/kg/day) + distilled water
- Group 3: L-NAME (40 mg/kg/day) + captopril (20 mg/kg/day)
- Group 4: L-NAME (40 mg/kg/day) + AESA (43 mg/kg/day)
- Group 5: L-NAME (40 mg/kg/day) + AESA (86 mg/kg/day)
- Group 6: L-NAME (40 mg/kg/day) + AESA (172 mg/kg/day)

The dose applied by the traditional healers was calculated to be 86 mg/kg/day. This dose was then divided and multiplied by two to obtain the other doses. All drugs were administered by gavage once a day. Body weight, blood pressure, and heart rate were weekly monitored, and at the end of the experiment, 24 h urine samples were collected, centrifuged at 3000 rpm for 15 min at 4°C (TGL-16M centrifuge, Loncare), and stored at -20°C for subsequent determination of nitric oxide (NO), creatinine, and proteins. After urine collection, animals were anesthetized by intraperitoneal injection of sodium thiopental (50 mg/kg). Blood samples were collected from the abdominal artery in heparinized tubes and centrifuged as described before, and the serum was collected and kept at -20°C for subsequent NO, creatinine, and protein quantification. The heart, kidney, and thoracic aorta were isolated, washed in saline, and weighed. The heart and the kidney were fixed in 10% buffered formalin for histological assessment.

#### 2.6.2. Determination of NO, Creatinine, and Proteins

Serum and urine NO content was quantified according to a previously described method (Fofié et al. [36]). Serum and urine creatinine was measured spectrophotometrically using Jaffe's reaction method [37]. Serum protein was quantified according to the Biuret method [38] while urinary protein excretion was assessed according to the Bradford method [39].

### 2.7. Histological Analysis

The heart and kidney were fixed in 10% formalin, embedded in paraffin, cut transversally into 4 *μ*m sections, and stained with hematoxylin and eosin (H&E). Structural abnormalities were visualized using a light microscope (DN-107T).

### 2.8. Statistical Analysis

Results were expressed as mean ± standard error of the mean (SEM), and the analysis was performed through GraphPad Prism 8.4.2 (GraphPad, USA). The differences of continuous variables among various groups were tested using one-way analysis of variance (ANOVA), followed by post hoc Tukey's multiple comparison test. Two-way ANOVA repeated measures followed by the Bonferroni post hoc test were used to analyze data with two variables. Data on NO release were analyzed with the Mann-Whitney test. Statistical significance was assigned for *p* values of less than 0.05.

## 3. Results

### 3.1. Phytochemical Composition of the Aqueous Extract from the Leaves of *S. abyssinica*

High-performance liquid chromatography gave a chromatogram showing many peaks with a retention time ranging 0 and 7 minutes.

**Figure 1:**
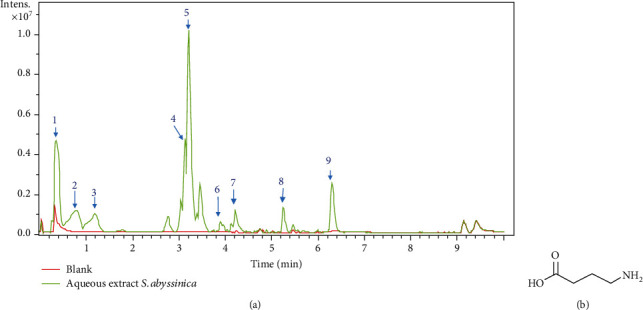
LC-MS chromatogram of aqueous extract from the leaves of *Stephania abyssinica* (a) and chemical structure of compound (1) *γ*-aminobutyric acid (GABA) (b).

The positive mode mass spectrum was used to determine the crude formula of nine compounds on the basis of ions and fragment ions corresponding to the peak observed at each retention time (TR) (Figure 1). Thus, the following molecular formulas were determined: (1) C_4_H_9_NO_2_ (TR: 0.4 min, *m*/*z*: 104.07), (2) C_19_H_15_N_3_O_3_ (TR: 0.8 min, *m*/*z*: 334.11), (3) C_21_H_23_N_3_O_2_ (TR: 1.2 min, *m*/*z*: 334.19), (4) C_24_H_27_N_3_O_3_ (TR: 3.1, *m*/*z*: 406.21), (5) C_24_H_25_N_3_O_3_ (TR: 3.2, *m*/*z*: 404.19), (6) C_28_H_37_NO_5_ (TR: 3.9 min, *m*/*z*: 468.27), (7) C_30_H_39_NO_5_ (TR: 4.3 min, *m*/*z*: 494.29), (8) C_41_H_36_O_4_ (TR: 5.3 min, *m*/*z*: 593.26), and (9) C_54_H_66_NO_8_ (TR: 6.3 min, *m*/*z*: 891.49) (Figure 1(a)).

Exception for compounds (1) and (8) which appeared, respectively, at 0.4- and 5.3-minute retention time, all other compounds were alkaloids. The positive mode mass spectrum shows an ion at *m*/*z* 104.07 [M + H]^+^ at the retention time of 0.4 minutes, with fragment ions at *m*/*z* 87.00, 138.56, 176.73, and 219.58 corresponding to the compound of the molecular formula C_4_H_9_NO_2_. After analysis and comparison of the spectra of the crude formulas and of the compounds already isolated from the genus Stephania and the Menispermaceae family, the compound (1) was identified as *γ*-aminobutyric acid (GABA) (Figure 1(b)), given that derivatives of GABA have been isolated from *Stephania rotunda* [40]. Research has not matched any other chemical formula with already isolated compounds from the genus Stephania or the Menispermaceae family.

### 3.2. Vasorelaxant Mechanisms of the Aqueous Extract of *S. abyssinica*

**Figure 2:**
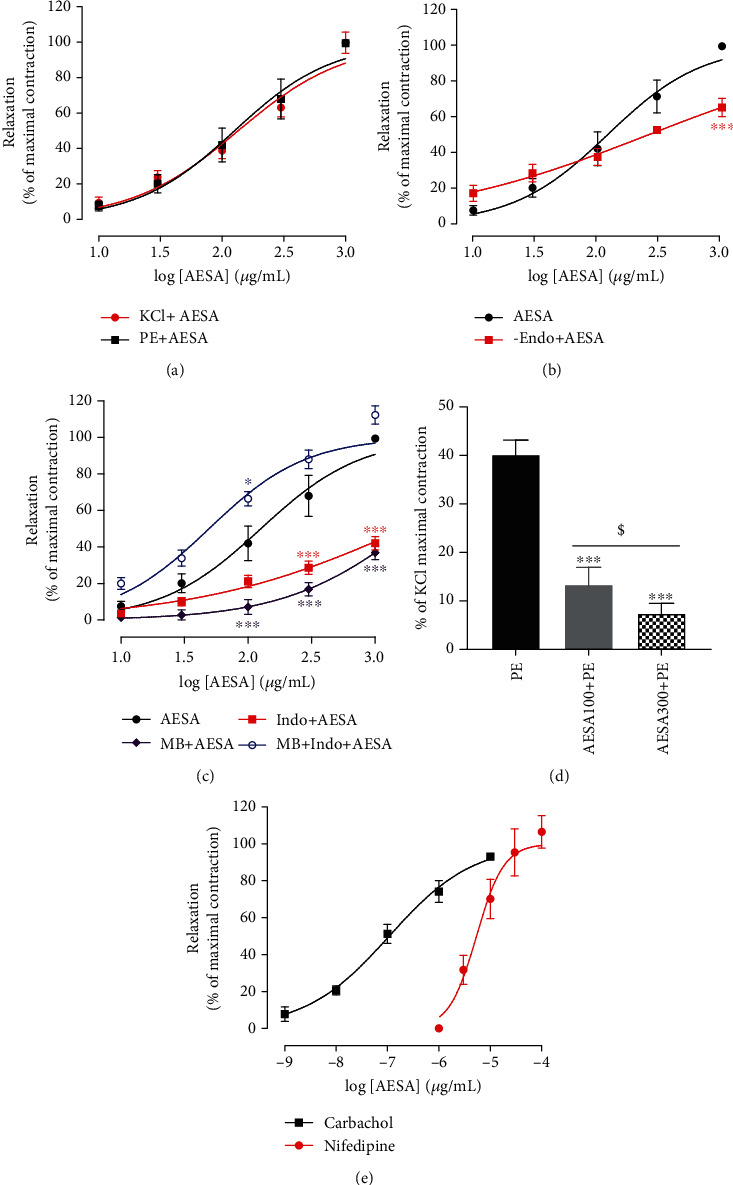
Effects of AESA on intact aortic rings precontracted with KCl or with phenylephrine (a), endothelium-denuded aortic rings precontracted with phenylephrine (b), intact aortic ring preincubated with indomethacin, methylene blue, and indomethacin + methylene blue (c), and on the intracellular Ca^2+^-release component of PE-induced contraction (d). Panel (e) presents the effects of reference substances (carbachol and nifedipine) on aortic rings precontracted with phenylephrine. Each point represents the mean ± SEM of six different experiments from six rats. Data were analyzed using ANOVA two-way with Bonferroni (a–c) or ANOVA one-way with Tukey's multiple comparison test (d). ^∗^*p* < 0.05 and ^∗∗∗^*p* < 0.001, significantly different compared to the effect of AESA without antagonists. ^$^*p* < 0.05 significant difference between the two concentrations.

The vasorelaxant effects of AESA were already demonstrated but the endothelial mediators involved in these effects as well as the participation of the intracellular calcium pathways were unknown. These sets of experiments were then undertaken to evaluate these aspects. Aortic rings with intact endothelium precontracted with KCl or phenylephrine (PE) were significantly and concentration-dependently relaxed by AESA (10-1000 *μ*g/mL) (Figure 2(a)) with respective EC_50_ of 134.70 and 126.00 *μ*g/mL and Emax of 99.72 ± 5.96 and 99.40 ± 1.85%. No significant difference was observed between the effects of AESA on KCl- and PE-induced contraction (Figure 2(a)). Aortic rings reacted normally to both KCl and PE after being exposed to extracts and washed.

The endothelium destruction significantly (*p* < 0.01) inhibited the AESA-induced relaxation in aortic rings precontracted with PE. EC_50_ was increased from 126.00 to 285.60 *μ*g/mL while Emax was reduced from 99.40 ± 1.85% to 65.07 ± 5.11% (Figure 2(b)).

The effects of AESA on PGI_2_/Cox and cGMP pathways were investigated by using, respectively, indomethacin and methylene blue as inhibitors. Results are presented in Figure 2(c). Pretreatment with indomethacin, a nonselective cyclooxygenase inhibitor (10 *μ*M), or with methylene blue, a guanylate cyclase inhibitor (10 *μ*M), significantly (*p* < 0.001) reduced the vasorelaxant response to AESA. EC_50_ increased from 126 to 1726 *μ*g/mL and 1850 *μ*g/mL while Emax was reduced from 99.40 ± 1.85% to 42.08 ± 3.68% and 36.94 ± 3.82%, respectively. None of the two substances was able to completely inhibit the effect of AESA. Surprisingly, the combination of indomethacin and methylene blue rather enhanced the vasorelaxant activity produced by AESA. EC_50_ was reduced from 126.00 to 49.27 *μ*g/mL while Emax was increased from 99.40 ± 1.85% to 112.25 ± 5.03%.

To determine whether AESA affects intracellular calcium release, its effect was also investigated on intracellular calcium release-induced contractions. The addition of PE in Ca^2+^-free Krebs evoked a contraction of about 40.14% of the maximal contraction induced with KCl in normal Krebs. Preincubation of aorta rings with AESA significantly (*p* < 0.001) and dose-dependently reduced the maximal contraction induced by PE. The effect of the AESA at the concentration of 300 *μ*g/mL was also significantly (*p* < 0.05) high as compared to the dose of 100 *μ*g/mL (Figure 2(d)).

### 3.3. Antihypertensive Activity of the Aqueous Extract from *S. abyssinica*

#### 3.3.1. Effect of AESA on Systolic and Diastolic Blood Pressure

**Figure 3:**
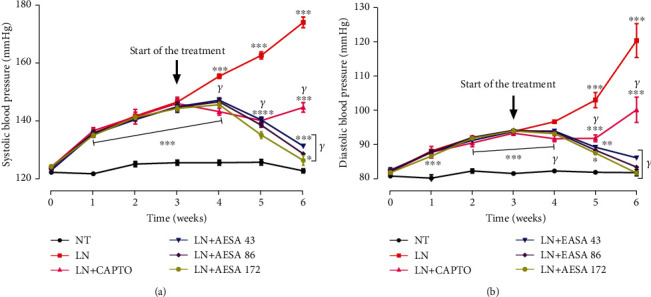
Effect of the leaf aqueous extract of *Stephania abyssinica* (AESA) on blood pressure of rats rendered hypertensive by chronic L-NAME administration. Values are expressed as mean ± SEM. *n* = 8; data were analyzed using ANOVA two-way with Bonferroni. ^∗∗∗^*p* < 0.001 significant difference compared to the normotensive control group; *^γ^p* < 0.001 significant difference compared to the L-NAME control group. NT = normotensive control; LN = L-NAME control; CAPTO = captopril; AESA 43, AESA 86, and AESA 172 = aqueous extract of *Stephania abyssinica* at the doses of 43, 86, and 172 mg/kg, respectively.

As shown in Figure 3, there was no significant difference in systolic (SBP) and diastolic blood pressure (DBP) among the different treatment groups at baseline. The chronic administration of L-NAME for 6 consecutive weeks induced a progressive and significant (*p* < 0.001) increase in SBP and DBP. SBP rose from 120.75 ± 0.84 mmHg in normal control rats to 174.25 ± 1.88 mmHg in L-NAME-treated rats. DBP reached 120.50 ± 4.96 mmHg in the L-NAME group as compared to 81.62 ± 0.92 mmHg in the normal control group. The administration of AESA induced a dose-dependent decrease in both SBP and DBP. SBP returned to nearly baseline value following AESA treatment while the DBP returned to the baseline value at doses of 86 and 172 mg/kg. Although the blood pressure of animals treated with captopril was significantly low (*p* < 0.001) compared to that of L-NAME-treated rats, it is important to notice that at the end of the experiment, a rebound effect was observed.

#### 3.3.2. Effect of the Aqueous Extract from *S. abyssinica* on Heart Rate

**Figure 4:**
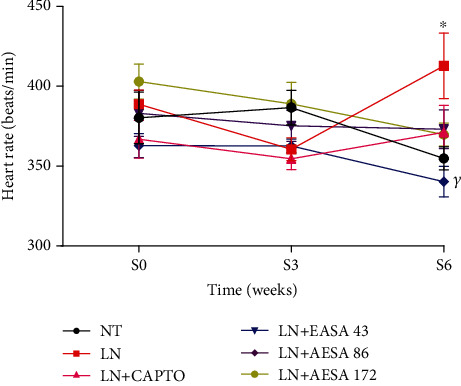
Effect of chronic administration of L-NAME alone and in combination with captopril (CAPTO) or the leaf aqueous extract of *Stephania abyssinica* (AESA) on heart rate. Values are expressed as mean ± SEM. *n* = 8; data were analyzed using ANOVA two-way with Bonferroni. ^∗^*p* < 0.05 compared to the normotensive control group. *^γ^p* < 0.001 significant difference compared to the L-NAME control group. NT = normotensive control; LN = L-NAME control; CAPTO = captopril; AESA 43, AESA 86, and AESA 172 = aqueous extract of *Stephania abyssinica* at 43, 86, and 172 mg/kg, respectively.

As shown in Figure 4, L-NAME administration induced an increase (*p* < 0.05) in heart rate compared to control (412.25 ± 20.46 vs. 354.87 ± 7.25 beats/min). All the treatments coadministered with L-NAME during the last three weeks evoked a decrease in heart rate. Captopril as well as AESA (43, 86, and 172 mg/kg) caused a reduction in heart rate (respectively, 371.12 ± 16.90, 373.12 ± 12.14, 340.25 ± 9.62, and 369.75 ± 7.20 beats/min). The effect of AESA (86 mg/kg) in lowering heart rate was significant (*p* < 0.001) compared to the LN group. There was no significant difference between the effects of the three doses of AESA used.

#### 3.3.3. Effect of the Aqueous Extract from *S. abyssinica* on the Body Weight and Organ Mass

As shown in Table 1, there was no significant difference in body mass gain. However, the gain in the L-NAME group was reduced by 32.78% as compared to the normal control. Only AESA at 43 and 86 mg/kg protected against this weight loss by up to 51.66% as compared to the L-NAME-treated group.

Chronic administration of L-NAME alone for 6 consecutive weeks induced a 24.56% increase in cardiac mass and a 27.26% augmentation of the left ventricular mass compared to the normal control group (Table 1). Only captopril was able to significantly (*p* < 0.05) reverse cardiac hypertrophy elicited by L-NAME.

Following L-NAME administration, the aorta mass significantly increased (*p* < 0.01) compared to the normal control. Captopril as well as AESA at doses of 43 and 86 mg/kg completely reversed L-NAME-evoked aorta hypertrophy. No significant change (*p* > 0.05) in kidney mass was observed throughout the experiment period (Table 1).

#### 3.3.4. Effect of the Aqueous Extract from *S. abyssinica* on Serum and Urine NO Levels

**Figure 5:**
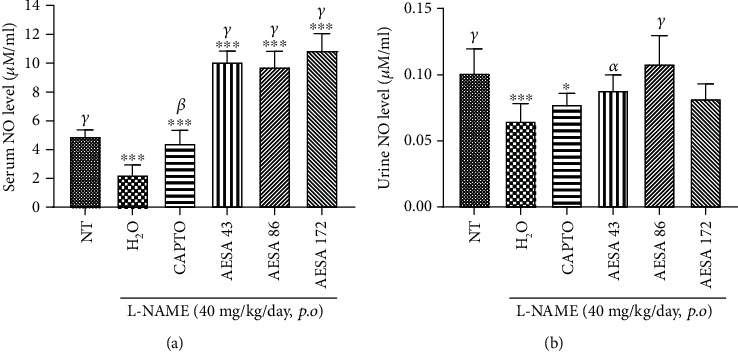
Effect of AESA on serum (a) and urine (b) nitric oxide concentration in L-NAME-induced hypertensive rats. Values are expressed as mean ± SEM. *n* = 8; data were analyzed using ANOVA one-way with Tukey's multiple comparison test. ^∗^*p* < 0.05 and ^∗∗∗^*p* < 0.001 compared to the normotensive control group. *^α^p* < 0.05, *^β^p* < 0.01, and *^γ^p* < 0.001 compared to the L-NAME (LN) control group. NT = normotensive control; LN = L-NAME control; LN + CAPTO = captopril; LN + AESA 43, LN + AESA 86, and LN + AESA 172 = aqueous extract of *Stephania abyssinica* at 43, 86, and 172 mg/kg, respectively.

As depicted in Figure 5, serum and urinary NO levels were significantly reduced (*p* < 0.001) when L-NAME was administered alone to animals. The coadministration of L-NAME with the plant extract significantly increased serum NO compared to both L-NAME and normal control groups (*p* < 0.001). On the same line, AESA at the doses of 43 and 86 mg/kg significantly (*p* < 0.05 and *p* < 0.01) corrected the effect of L-NAME on urinary NO.

#### 3.3.5. NO Content of the Aqueous Extract from *S. abyssinica*

**Figure 6:**
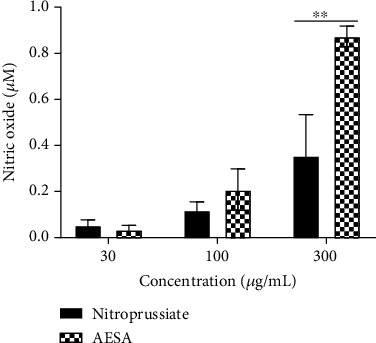
NO content of the aqueous extract of *S. abyssinica*. Values are expressed as mean ± SEM. *N* = 4. Data were analyzed by paired concentration using the Mann-Whitney test. ^∗∗^*p* < 0.01 significant difference between the two substances.

To better understand the pharmacological effects of the plant extract, we assessed whether it could contain or release nitric oxide. As shown in Figure 6, titration revealed that AESA has a high content of nitric oxide. AESA released NO in a concentration-dependent manner and released more NO than sodium nitroprusside at equivalent concentrations.

#### 3.3.6. Effect of the Aqueous Extract of *S. abyssinica* on Kidney Function

Table 2 shows the effects of *S. abyssinica* aqueous extract on some parameters of kidney function. It can be observed that L-NAME administration did not affect rats' serum proteins compared to the normotensive control group. In the same line, captopril and AESA treatments did not influence serum protein level except the dose 172 mg/kg which induced a significant (*p* < 0.05) serum protein increase. On the other hand, rats that receive L-NAME alone have an increased protein excretion, high serum creatinine levels, and reduced creatinine excretion and GFR while the urine output was unchanged. Captopril reduced the effect of L-NAME on proteinuria and increased creatinine excretion in urine as well as GFR. The plant extract exacerbated the effect of L-NAME on proteinuria and GFR, especially at the dose of 172 mg/kg.

### 3.4. Effects of the Aqueous Extract of *S. abyssinica* on Histology of the Heart and Kidney

**Figure 7:**
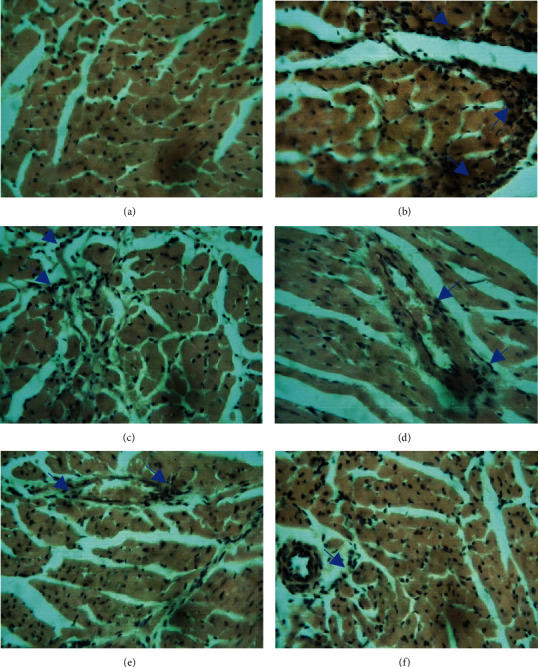
Photomicrographs of histopathological changes in the heart: (a) control, no observable changes; (b) L-NAME alone, showing important inflammatory cell infiltration (blue arrows); (c) L-NAME + captopril; (d–f) L-NAME + AESA at respective doses 43, 86, and 172 mg/kg, showing reduced inflammatory cell infiltration. H&E, mag 400x.

The inspection of the heart histopathological slices showed normal structure of the myocardium in rat of the normotensive group (Figure 7(a)) while leukocyte infiltration was observed in the L-NAME hypertensive group (Figure 7(b)). Captopril and AESA- (43, 86, and 172 mg/kg) treated groups showed a significant reduction in inflammatory cell infiltration (Figures 7(c)–7(f)).

**Figure 8:**
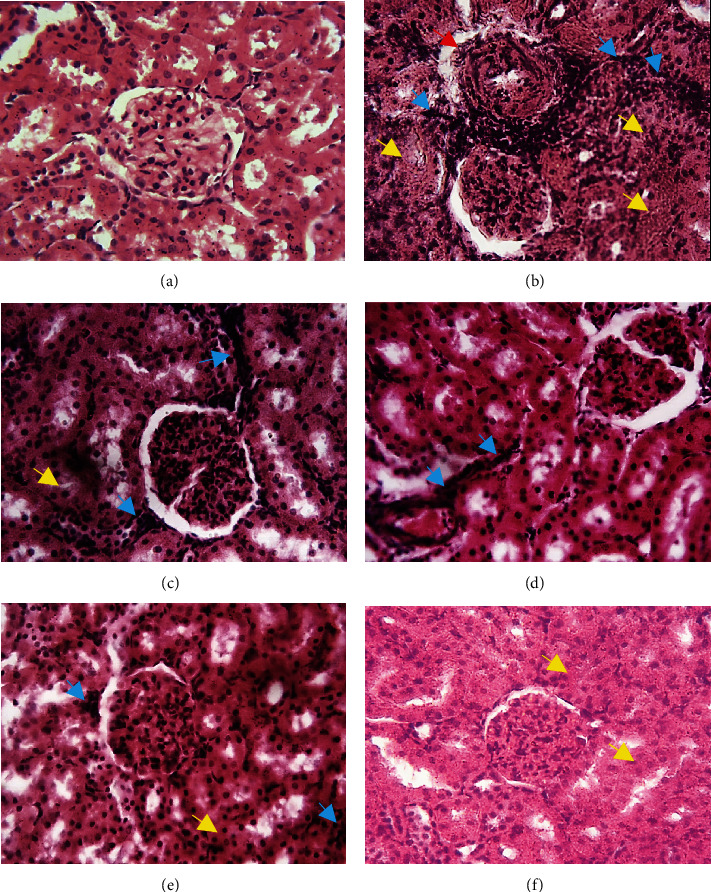
Representative photomicrographs of kidneys from the experimental rats. (a) Kidneys of control rats showing normal kidney histological architecture. (b) Kidneys from L-NAME-treated rat leukocyte infiltration (blue arrows), arterial wall thickening (red arrow), and tubular disorganization (yellow arrow). (c) Kidneys from L-NAME + captopril and (d, e) L-NAME + AESA- (43 and 86 mg/kg) treated groups showing attenuated infiltrations and lesions. Although rats receiving L-NAME + AESA at 172 mg/kg showed almost no infiltration, the histological architecture was strongly affected. H&E, mag 400x.

Figure 8 depicts representative photomicrographs of the kidneys from the normotensive control and various treatment groups. No histopathological change was observed in the kidneys of the normotensive group (Figure 8(a)). However, the kidneys from L-NAME-induced hypertensive rats showed histopathological lesions including leukocyte infiltration, arterial wall thickening, and tubular disorganization (Figure 8(b)). These renal histopathological lesions induced by the L-NAME chronic administration were attenuated in the captopril and AESA-treated group (Figures 8(c)–8(f)).

## 4. Discussion

In a previous study, we showed that AESA possesses endothelium nitric oxide-independent vasorelaxant effects on the isolated rat thoracic aorta but neither propranolol, tetraethylammonium, nor glibenclamide could completely block the vasorelaxant activity of the extract. More, AESA was unable to completely suppress extracellular calcium-induced vascular contraction [14]. We then hypothesized that AESA may possess additional mechanisms that might trigger its vasorelaxant activity. Besides, AESA is used by local populations to treat arterial hypertension. It was subsequently thought that its potential antihypertensive effect may be supported by the upmentioned vasodilating properties. The present work was undertaken to evaluate this hypothesis. AESA relaxant effect was significantly affected by the removal of the vascular endothelium and pretreatment with indomethacin, methylene blue, or the combination of both antagonists. In addition, AESA significantly inhibited the intracellular calcium-induced vascular contraction and reduced L-NAME-induced arterial hypertension.

It was observed in the present study that AESA similarly relaxed aortic rings precontracted with KCl or PE. This result is in accordance with previous observation [14] and confirmed that AESA is capable to relax both voltage-operated calcium channels and receptor-operated calcium channels. Vascular relaxation depends on two main pathways, the release of endothelial relaxing factors or the direct effect on the vascular smooth muscles. To evaluate whether the endothelium mediators contribute to the vasorelaxant effect of AESA, the plant extract was tested on endothelium-denuded aortic ring precontracted with PE. The removal of the endothelium partially inhibited the vasorelaxant effect of AESA suggesting the implication of endothelium relaxing factors in the vasorelaxation induced by AESA.

Mechanisms by which plant extracts or natural products can induce endothelium-dependent vasorelaxation involve nitric oxide (NO), prostacyclin (PGI_2_), or endothelial-derived hyperpolarizing factor [41, 42]. Among these endothelium-derived relaxing factors, only NO and PGI_2_ are well characterized [43]. In a previous study, it was shown that endothelial NO did not mediate AESA-evoked vasorelaxation [14]. So we hypothesized that PGI_2_ might be responsible for the endothelium-mediated AESA relaxation. PGI_2_ is primarily synthesized from arachidonic acid catalyzed by cyclooxygenase (COX) [42]. The incubation of intact aortic rings with indomethacin, a COX inhibition, before the AESA challenge significantly reduced the vasorelaxant activity of AESA suggesting that PGI_2_ mediates AESA-induced vasorelaxation of aortic rings.

Even with the strong inhibitory effect of indomethacin, AESA was still able to elicit about 40% relaxation, suggesting other mechanistic pathways. AESA was then tested in the presence of methylene blue, an inhibitor of soluble guanylate cyclase. The activation of soluble guanylate cyclase results in the generation of cyclic guanosine monophosphate (cGMP). The increase in intracellular cGMP concentration activates cGMP-dependent protein kinase (PKG), which causes vasorelaxation via the modulation of Ca^2+^ channels as well as by decreasing the Ca^2+^ sensitivity of the vascular smooth muscle contractile proteins [44]. Unexpectedly, the AESA effect was strongly reduced by the methylene blue, suggesting the involvement of the sGC/cGMP pathway in the vasorelaxation activity of AESA. Moreover, when the aortic rings were preincubated with both indomethacin and methylene blue, the combination of these two inhibitors highly potentiated the vasorelaxant activity of AESA. This suggests that when PGI_2_ synthesis and soluble guanylate cyclase are both inhibited, AESA induced vasorelaxation by other mechanisms. In a previous study, we demonstrated that glibenclamide (an ATP-sensitive K^+^ channel blocker) greatly reduced AESA-induced aortic muscle relaxation [14]. Thus, the activation of K_ATP_ channels resulting in membrane hyperpolarization might explain the COX and cGMP-independent vasorelaxation observed in this study. Some studies have shown that when basal NO synthesis is blocked, relaxation is due to a combination of both potassium channel and guanylyl cyclase activation [45]. A charybdotoxin-sensitive K^+^ channel has found to be also implicated in the vasorelaxation with the NO donors [46]. NO has been shown to stimulate BK_Ca_ channels independently of cyclic GMP in vascular smooth muscle cells [47–49]. It was somehow contradictory that the sGC/cGMP pathway contributes to the vasorelaxant effect of AESA which has been considered endothelial NO-independent. To better understand this, we quantified the NO content/release by AESA and realized that it is highly rich in NO. NO donors have been found to evoke smooth muscle cell hyperpolarization through activation ATP-sensitive potassium channels (K_ATP)_ [50, 51], voltage-activated potassium channels (K_V_) [52, 53], inwardly rectifying potassium channels (Kir) [54, 55], two-pore-domain potassium channels (K_2_P) [56], and BK_Ca_ [57, 58]. Therefore, AESA might be a direct NO donor and could, therefore, exert a direct hyperpolarizing mechanism on smooth muscle cells.

Vascular smooth muscle contraction is triggered by an increase in intracellular Ca^2+^ contraction, resulting from an increase in calcium influx and/or intracellular stores' calcium release. Furthermore, this is part of the vasorelaxant mechanism of NO. In the present study, the effect of AESA on intracellular calcium was investigated. Preincubation of aortic rings with AESA significantly reduced PE-induced contraction. This result suggests that AESA may cause the vasorelaxation of aortic smooth muscle by also inhibiting the release of Ca^2+^ from intracellular stores.

It is well-known that arterial hypertension (AHT) is associated with vascular changes characterized by endothelial dysfunction, increased vascular contraction, and arterial remodeling [59, 60]. Taking into account the vasorelaxant effect of AESA as demonstrated in this study, we hypothesized that this extract may improve hemodynamic, functional, and structural abnormalities in a rat model of hypertension. Thus, the antihypertensive effect of AESA was examined *in vivo*, using the L-NAME-induced hypertension model in rats, which provides a reliable model of hypertension with pronounced target organ damages that mimic AHT seen in humans.

In this study, the chronic oral administration of L-NAME was associated with a significant rise in BP and pulse rate compared with the normotensive control rats, validating the induction of hypertension. Administration of AESA and captopril (a control hypotensive drug) for three weeks caused a significant decline in BP and pulse rate in the hypertensive rats. Various studies have suggested that chronic NOS inhibition with L-NAME increases sympathetic activity release and heart rate [42, 61]. Therefore, sympathoexcitation suppression and vasodilation could be a possible mechanism of the significant decrease in arterial pressure and heart rate induced by AESA in L-NAME-treated rats.

L-NAME treatment significantly reduced serum NO, urinary creatinine and NO, and glomerular filtration rate (GFR) but increased serum creatinine and urinary proteins. AESA significantly increased the bioavailability of NO; this may be related to its high content in nitric as observed in in vitro testing. These results further indicate that the antihypertensive effect of AESA is at least partially related to the increase in NO bioavailability and, thus, to its vasorelaxant activity. Although captopril and the lower doses of AESA tended to reverse the renal effects of L-NAME, the plant extract at the dose of 172 mg/kg worsens the parameters. These findings indicate that the higher dose of AESA although very efficient on AHT might be harmful for kidney function. Indeed, the histological analysis of the kidney revealed that AESA at 43 and 86 mg/kg corrected the renal alterations induced by L-NAME. Though there was a significant reduction of inflammatory cell infiltration in the kidney of the rats receiving AESA at 172 mg/kg, the microarchitecture of the organ was disorganized.

L-NAME-induced pressure overload is associated with cardiac and arterial remodeling and stiffening which may initiate pathological changes in the cardiovascular and renal systems. L-NAME administration increased the heart and aorta masses. This is a well-known phenomenon in L-NAME hypertensive rats [62]. AESA tended to reduce the aorta mass but not that of the heart. This result is a bit hard to understand given that the same mediators are involved in the remodeling of both tissues. Besides, it has been demonstrated that increased NO bioavailability reduces the L-NAME's proliferative effect in the heart and aorta [63, 64]. Surprisingly, AESA increased the NO availability but failed to reduce L-NAME-induced cardiac hypertrophy.

The histopathological examination of the heart of the L-NAME hypertensive group revealed pronounce leukocyte infiltration. Various studies demonstrated that NO-deficient hypertension by L-NAME resulted in marked cardiac inflammation due to a significant increase in the cardiac density of macrophages and T-cells that produce several cytokines [65]. AESA administration reduced leukocyte infiltration. These findings suggest its cardioprotective effect. Nevertheless, the plant extract was unable to reduce the cardiac mass.

## 5. Conclusion

These findings showed that AESA exerted effective vasorelaxant effects in isolated rat thoracic aorta rings. This vasorelaxation could be mediated partly by an endothelium-dependent mechanism involving the prostacyclin pathway and partly by an endothelium-independent mechanism through direct activation of sGC/cGMP and K^+^ channels in vascular smooth muscle, leading to inhibition of Ca^2+^ influx from the extracellular milieu and IP_3_-sensitive intracellular Ca^2+^ release. The vasorelaxant properties of AESA *in vitro* may be translated to a potent antihypertensive effect *in vivo*. Therefore, AESA may be a valuable drug candidate for arterial hypertension. However, caution should be taken with higher doses as they may induce kidney damage.

## Figures and Tables

**Table 1:** Effect of L-NAME alone and in combination with captopril (CAPTO) or the leaf aqueous extract of *Stephania abyssinica* (AESA) on relative organs' mass.

	NT	LN	LN + CAPTO	LN + AESA 43	LN + AESA 86	LN + AESA 172
Body weight gain (g)	42.00 ± 4.62	28.25 ± 2.03	26.38 ± 4.32	39.75 ± 5.73	45.13 ± 8.11	23.63 ± 3.81
Heart (mg/cm)	222.00 ± 8.53	242.00 ± 3.53	206.00 ± 7.09*^α^*	251.00 ± 7.73	239.00 ± 9.15	228.00 ± 9.40
Left vent. (mg/cm)	158.00 ± 4.03	178.00 ± 3.37	154.00 ± 6.77	186.00 ± 6.13	171.00 ± 8.61	176.00 ± 6.32
Aortae (mg/cm)	20.00 ± 1.21	27.60 ± 1.96^∗∗^	17.60 ± 1.10*^α^*	21.60 ± 1.65	19.10 ± 1.52	22.6 ± 1.35
Kidneys (mg/cm)	458.00 ± 20.00	473.00 ± 39.5	493 ± 52.10	556.00 ± 70.80	473.00 ± 19.2	488.00 ± 32.7

Values are expressed as mean ± SEM. The relative mass was calculated by dividing the absolute mass by the tibia length. *n* = 8; data were analyzed using ANOVA one-way with Tukey's multiple comparison test. ^∗^*p* < 0.05, ^∗∗^*p* < 0.01, and ^∗∗∗^*p* < 0.001 compared to the normotensive control group; *^α^p* < 0.05 compared to the L-NAME (LN) control group. NT = normotensive control; LN = L-NAME control; LN + CAPTO = captopril; LN + AESA 43, LN + AESA 86, and LN + AESA 172 = aqueous extract of *Stephania abyssinica* at 43, 86, and 172 mg/kg, respectively.

**Table 2:** Effect of AESA on kidney function in L-NAME-induced hypertensive rats.

	NT	LN	LN + CAPTO	LN + AESA 43	LN + AESA 86	LN + AESA 172
Serum protein (nmol/*μ*L)	13.44 ± 0.17	13.84 ± 0.57	13.18 ± 0.25	13.62 ± 0.61	32 ± 0.33	15.13 ± 0.45^∗^
Urine protein (nmol/*μ*L)	7.42 ± 2.74	19.27 ± 4.16	13.09 ± 2.84	24.34 ± 6.63	31.68 ± 3.94^∗∗^	34.940 ± 3.91^∗∗∗^
Serum creatinine (*μ*g/mL)	3.99 ± 0.14	7.094 ± 0.12^∗∗∗^	4.18 ± 0.33*^γ^*	5.73 ± 0.35	4.96 ± 0.52*^β^*	3.74 ± 0.42*^γ^*
Urine creatinine (*μ*g/mL)	15.13 ± 1.11	8.25 ± 0.56^∗∗∗^	27.97 ± 0.79^∗∗∗^*^γ^*	13.71 ± 0.95	26.55 ± 1.07^∗∗∗^*^δ^*	6.89 ± 0.47^∗∗∗^*^γ^*
Urine volume (mL/24 h)	22.38 ± 3.58	19.18 ± 2.22	18.86 ± 2.38	20.79 ± 2.93	12.49 ± 0.68	19.29 ± 2.36
GFR (*μ*L/min)	41.60 ± 4.09	20.30 ± 1.96^∗^	85.8 ± 1.96^∗∗∗^*^γ^*	29.3 ± 2.90	22.1 ± 4.00	17.7 ± 2.52^∗^

Values are expressed as mean ± SEM. *n* = 8; data were analyzed using ANOVA one-way with Tukey's multiple comparison test. ^∗^*p* < 0.05, ^∗∗^*p* < 0.01, and ^∗∗∗^*p* < 0.001, compared to the normotensive control group; *^α^p* < 0.05, *^β^p* < 0.01, and *^γ^p* < 0.001, compared to the L-NAME (LN) control group. NT = normotensive control; LN = L-NAME control; LN + CAPTO = captopril; LN + AESA 43, LN + AESA 86, and LN + AESA 172 = aqueous extract of *Stephania abyssinica* at 43, 86, and 172 mg/kg, respectively.

## Data Availability

The data used and analyzed in this study are available from the corresponding author on reasonable request.
